# Effects of L-glutamic acid on wheat starch structure and functionality: multiscale characterization and molecular dynamics simulations

**DOI:** 10.1016/j.fochx.2026.104267

**Published:** 2026-07-31

**Authors:** Gang Li, Song Shen, Ke Wang, Kaiyong Fu, Jialian Wei, Heqin Wei, Xun Zhu, Chunyan Li, Cheng Li

**Affiliations:** College of Agriculture, Shihezi University/Key Laboratory of Oasis Ecological Agriculture, Shihezi 832003, China

**Keywords:** Glutamic acid, Metabolomics, Starch, Structural properties, Functional properties, Molecular dynamics simulation

## Abstract

L-Glutamic acid (L-Glu) is central to carbon–nitrogen metabolism, yet its ability to improve starch quality by regulating grain metabolism and by directly modulating starch structure remains unclear. In this study, we integrated a field experiment with an in vitro experiment and combined untargeted metabolomics, microscopy, structural characterization, physicochemical and processing property analyses, and molecular dynamics simulations. It was found that under drought, L-Glu spraying reprogrammed the grain metabolome, with pathway enrichment in aminoacyl-tRNA biosynthesis and starch/sucrose metabolism. In vitro, L-Glu increased the full width at half maximum of the Raman band at 480 cm^−1^ and altered the FTIR absorbance ratio of 1047/1022 cm^−1^. L-Glu enhanced in vitro digestibility and reduced swelling power and paste transparency, while decreasing setback viscosity, suggesting a potential inhibition of short-term starch retrogradation; however, freeze–thaw stability was reduced. Microscopy revealed a more disordered gel network after L-Glu addition. Simulations showed that L-Glu formed 1.47–2.18 persistent hydrogen bonds with amylose chain per system and extensive hydrogen bonds with water.

## Highlights

• Foliar-applied L-Glu markedly altered grain metabolism under drought stress.
• L-Glu addition in vitro greatly enhanced starch digestibility.
• In vitro addition of L-Glu significantly regulated starch retrogradation.
• L-Glu forms 1–2 stable intermolecular hydrogen bonds with starch chains per system.
• L-Glu can form extensive hydrogen bonds with water.

## Graphical abstract

**Figure:**
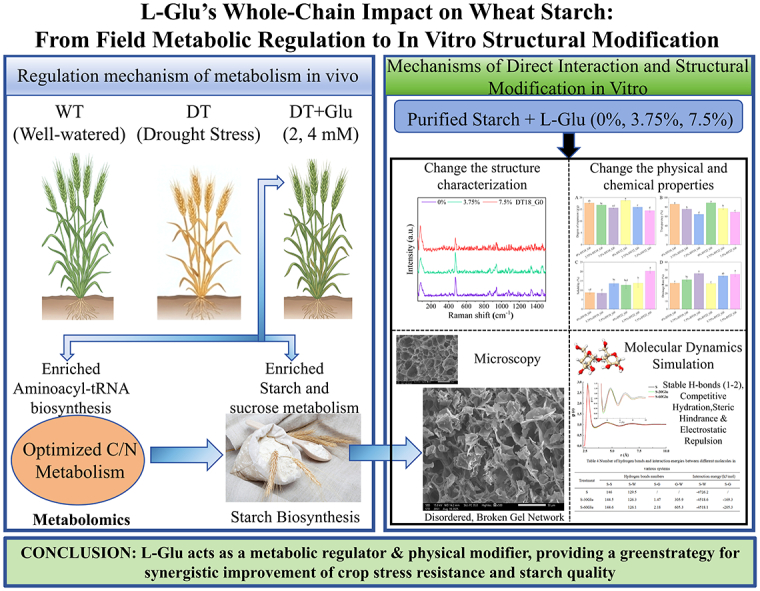

## 1 Introduction

Wheat (*Triticum aestivum* L.) is a major staple crop worldwide, and both its yield and end-use quality are central to global food security (Curtis & Halford, 2014; Li et al., 2015). Starch is the predominant component of wheat grain, and its multiscale structure largely determines the processing performance and sensory quality of wheat-based foods (Zhang et al., 2022). Post-anthesis drought stress reduces yield and perturbs metabolism and physiology in developing grains, often leading to inferior starch quality (Li et al., 2024). In addition, native starch presents practical limitations, including high paste viscosity and poor freeze–thaw stability, which can complicate processing and impair texture in starch-based products (Dartois et al., 2010; Singh et al., 2007). These challenges motivate the development of effective starch-modification strategies.

Metabolomics provides a systems-level approach to dissect plant responses to abiotic stress. Drought stress has been shown to reshape the grain metabolome, particularly amino acids, organic acids, and sugars, and these shifts are closely linked to starch biosynthesis (Lv et al., 2022). Beyond serving as building blocks for proteins, amino acids also act as signaling molecules and metabolic regulators during stress responses. For example, L-Glu has been reported to alleviate chilling injury in plum fruit by modulating reactive oxygen species homeostasis, the GABA shunt, and energy metabolism, accompanied by changes in metabolites and gene expression related to amino acid and secondary metabolite biosynthesis (Hou et al., 2024; Hou et al., 2025). L-Glu is also a natural food constituent and has applications as a food additive and nutritional supplement in certain contexts (Gałkowska & Juszczak, 2019).

Charged amino acids can also directly influence starch structure and functionality (Cui et al., 2014). Glycine, glutamic acid, lysine, and related amino acids have been reported to modulate the physicochemical, rheological, and thermal properties of starches from various botanical sources, including potato, sweet potato, rice, corn, and wheat (Chen et al., 2015; Lockwood et al., 2008; Lu et al., 2012; Majzoobi et al., 2011; Wan et al., 2017). As a central node in amino acid metabolism, L-Glu lies at the intersection of the tricarboxylic acid (TCA) cycle and nitrogen metabolism (Li et al., 2024; Liao et al., 2022), and it may influence starch biosynthesis and higher-order structure formation by altering carbon–nitrogen fluxes and enzyme activities. Although electrostatic interactions have been proposed to underlie amino acid–starch effects (Xu et al., 2022), mechanistic interpretation remains largely based on bulk measurements, and molecular-level evidence is still limited.

Molecular dynamics (MD) simulations are a powerful approach for probing interactions between biomacromolecules and small molecules. They can capture molecular trajectories, conformational changes, and interaction details at atomic resolution, thereby providing molecular-level mechanistic insight into macroscopic observations (Śledź & Caflisch, 2018). Using this technique, previous studies have elucidated hydrogen-bonding patterns and conformational regulation between proteins and starch chains, demonstrating its ability to quantify starch–protein interaction energies and changes in the hydration layer (Chen et al., 2019). Our previous work showed that, under post-anthesis drought stress, foliar application of L-Glu promoted starch accumulation in wheat grains and mitigated the negative effects of drought on starch quality across multiple structural scales, thereby improving its functional properties (Li et al., 2025). Although existing studies have separately focused on drought-induced changes in wheat physiology or alterations in starch characteristics, systematic efforts linking upstream metabolomic responses to downstream starch structure–function traits remain limited. In particular, it remains unclear how L-Glu integrates drought-induced metabolic reprogramming as a metabolic regulator and, as an exogenous additive, triggers cascading effects on starch from crystalline structure and ultrastructure to macroscopic functionality. Moreover, most available studies remain at the level of macroscopic characterization. Direct atomic-level evidence is still lacking regarding the interaction modes between L-Glu and starch molecules (e.g., hydrogen bonding, steric hindrance, and electrostatic effects) and the molecular origins by which such interactions drive starch structural reconstruction, which limits a deeper understanding of its modification mechanism.

Based on preliminary investigations into the regulation of cereal starch biosynthesis by amino acids (Li et al., 2025), the present study proposes the core hypothesis that foliar application of L-Glu under post-anthesis drought stress can significantly alter the metabolic profile of wheat grains, thereby constructing an optimized metabolic microenvironment for starch biosynthesis. Furthermore, from the perspective of molecular action mechanisms, non-covalent interactions between L-Glu and starch molecules represent the key intrinsic mechanism mediating directional changes in the structural and functional characteristics of starch. This also renders L-Glu a promising novel wheat starch physicochemical properties improver, applicable to the customized regulation of starch functionalities in the food industry. To verify the above hypothesis, this study integrated field foliar application trials with in vitro starch blending assays to systematically investigate the regulatory effects of L-Glu on wheat, spanning from grain metabolism to the structural and functional characteristics of starch. For the first time, this study establishes a direct link between upstream grain metabolic responses and downstream starch structural and functional traits in wheat, and provides robust theoretical and mechanistic support for the potential application of L-Glu as a physicochemical property improver to directionally regulate the functionalities of wheat starch for food applications.

## 2 Materials and methods

### 2.1 Experimental design

#### 2.1.1 Field experiment design

The winter wheat varieties used in the field experiment were Xindong 18 (XD18) and Xindong 22 (XD22). L-Glu was purchased from Beijing Coolaber Technology Co., Ltd. (Cat# cg5771-100 g), with the chemical formula C_5_H_9_NO_4_, appearing as a colorless crystal, and a molecular weight of 147.13. The field experiment was conducted during the 2023–2024 growing season at the Teaching and Experimental Farm of Shihezi University. Two irrigation treatments were set up: (1) Well-watered treatment (WT): normal irrigation was applied from sowing to anthesis and from anthesis to maturity. (2) Drought treatment (DT): irrigation was the same as WT from sowing to anthesis, but completely withheld from anthesis to maturity.

Foliar spraying of L-Glu solutions was performed on stems, leaves, and spikes at heading and flowering stages. The WT group was sprayed with 0 mM L-Glu (The aim of this study was to investigate the regulatory and alleviating effects of exogenous glutamate on drought stress damage, so the WT treatment was only sprayed with water). The DT group included three L-Glu concentrations: 0 mM, 2 mM, and 4 mM. The four field treatments were designated as follows: WT-0 mM L-Glu (WT_G0), DT-0 mM L-Glu (DT_G0), DT-2 mM L-Glu (DT_G2), and DT-4 mM L-Glu (DT_G4). The specific foliar application method and experimental design followed the procedures described in our previous studies (Li et al., 2024; Li et al., 2025).

#### 2.1.2 Laboratory experiment design

We weighed out 10 g of wheat grains of XD18 and XD22 under the DT_G0 treatment (from 2.1.1, drought stress without foliar L-Glu). Starch extraction and purification were subsequently conducted after the grains were milled into powder. The flour was transferred into a 50 mL centrifuge tube, and distilled water was added to a final volume of 50 mL, and the mixture was repeatedly shaken and centrifuged at 4000×*g* for 10 min. This process was repeated three times. The supernatant was discarded, and the resulting precipitate was layered onto 80% (*w*/*v*) CsCl solution. After centrifugation, the starch layer was washed three times with washing buffer (62.5 mmol·L^−1^ Tris-HCl, pH 6.8; 10 mmol·L^−1^ EDTA; 4% [*w*/*v*] SDS), deionized water, and acetone, respectively, and then air-dried naturally.

L-Glu was added to starch at 0, 3.75, and 7.5% (*w*/w, starch basis), respectively. Specifically, 0, 0.075, or 0.15 g L-Glu was first dissolved in 100 mL distilled water to facilitate homogeneous mixing, after which 2.0 g purified starch was added, respectively. The mixture was shaken at 220 rpm (25 °C) for 4 h, dried in a convection oven at 35 °C for 48 h, and ground to obtain L-Glu–starch composite samples.

#### 2.1.3 Molecular dynamics simulation of glutamic acid–starch systems

Molecular dynamics (MD) simulations were conducted using GROMACS 2021.3. The simulation systems were designed as follows: a single-helical amylose chain containing 55 glucose units (provided by Prof. Tao Feng, University of Shanghai for Science and Technology) was used as the starch model. Three simulation systems were constructed: Control group (S): starch in water without L-Glu (simulation box: 4.3 × 4.3 × 10.7 nm). Low-concentration group (S-30Glu): one starch chain with 30 randomly distributed L-Glu molecules (simulation box: 7.8 × 7.8 × 10.7 nm). High-concentration group (S-60Glu): one starch chain with 60 randomly distributed L-Glu molecules (simulation box: 8.5 × 8.7 × 10.7 nm). The structure and topology files of L-Glu were generated using the LigParGen server. Both starch and L-Glu were described with the OPLS-AA all-atom force field. All systems were solvated using the simple point charge (SPC) water model, and sodium ions were added to ensure electroneutrality.

Energy minimization was conducted using the steepest descent algorithm for 10,000 steps until the maximum force was ≤1000 kJ/mol/nm. The systems were then equilibrated for 1 ns under the constant number of particles, pressure, and temperature (NPT) ensemble (298.15 K, Nosé–Hoover thermostat; 1 bar, Parrinello–Rahman barostat). After the density stabilized, production simulations were performed for 50 ns under the NVT ensemble using the leap-frog integrator with a 2 fs time step. A 1.0 nm cutoff was applied for short-range interactions; long-range electrostatics were computed using the particle-mesh Ewald (PME) method; and bonds involving hydrogen were constrained using linear constraint solver (LINCS).

All molecular dynamics (MD) simulation results in this study, including root mean square deviation (RMSD), dihedral angle distribution, radial distribution function (RDF), hydrogen bond count, atomic density distribution, and L-Glu aggregation state analysis, were statistically analyzed based on data from the equilibrium phase of a single 50 ns simulation trajectory. To ensure the representativeness of the results, the system was confirmed to have reached a stable equilibrium state in the late stage of the simulation via RMSD curve prior to trajectory selection. Future work will further conduct multiple independent replicate simulations to enhance the statistical reliability of the findings.

### 2.2 Determinations and methods

#### 2.2.1 Untargeted metabolomics analysis of field-grown grains

Wheat grains at 35 days after anthesis from each treatment in the field experiment described in Section 2.1.1 were collected and subjected to untargeted metabolomics analysis by Nanjing Paisennuo Gene Technology Co., Ltd. Metabolites were extracted using a precooled methanol-acetonitrile-water mixture (2:2:1, v/v/v). After grinding, ultrasonication, and refrigerated centrifugation, the supernatant was collected and filtered. Metabolite separation was performed using an ACQUITY UPLC HSS T3 column with water containing 0.1% formic acid and acetonitrile containing 0.1% formic acid as the mobile phases under a gradient elution program. Mass spectrometry was conducted on a Thermo Orbitrap Exploris 120 instrument using data-dependent acquisition (DDA) in both positive and negative ion modes. Raw data were processed in MS-DIAL for peak extraction, alignment, and metabolite identification. Compound annotation was completed based on the company's in-house database and multiple public databases (e.g., HMDB and LIPID MAPS), followed by quality control and normalization.

#### 2.2.2 Protease hydrolysis of starch granules and confocal laser scanning microscopy

Starch granules (200 mg) from the WT_G0 and DT_G0 treatments in the field experiment (Section 2.1.1) were suspended in 2 mL of enzymatic digestion buffer containing 400 U protease XIV (Sigma, Lot#051M1894V, USA), respectively. The digestion buffer consisted of 0.02% (w/v) sodium azide, 0.05 mol/L sodium acetate, and 1 mmol/L calcium chloride (pH 7.5). The suspension was gently shaken at 4 °C for 24 h, followed by centrifugation at 5000 ×*g* for 10 min. The digested starch granules were collected, washed with 2 mL distilled water, centrifuged again at 5000 ×*g* for 10 min, and air-dried. Then, 50 mg of the dried digested starch granules were suspended in 2 mL of merbromin solution in methanol (0.1%, w/v) and gently shaken at room temperature for 4 h. The stained starch granules were collected, washed with 2 mL ethanol, and centrifuged at 5000×*g* for 10 min; this washing step was repeated three times. The granules were then collected and air-dried.

A small amount of stained starch granules was placed on a glass slide, one drop of glycerol (20%, v/v) was added, and a temporary slide was prepared. Images were acquired using a confocal laser scanning microscope (Zeiss, Oberkochen, Germany) at an excitation wavelength of 488 nm (Li, Fu, et al., 2025; Li et al., 2025).

#### 2.2.3 Alpha-amylase hydrolysis of L-Glu-starch mixtures and determination of reducing sugar concentration

For each L-Glu-starch mixed sample prepared in the in vitro experiment (Section 2.1.2), 25 mg was weighed and mixed with 1 mL sodium acetate buffer (0.1 mol/L, pH 4.8) containing 360 U alpha-amylase (Solarbio, A8750). The reaction was carried out at 37 °C and 200 rpm for 36 h. Then, 50 μL HCl (1 mol/L) was added, and the pH was adjusted to 7.0 using NaOH (1 mol/L) to terminate the enzymatic reaction. Next, 0.5 mL of 95% ethanol was added, followed by centrifugation at 1500 rpm for 10 min. The supernatant was then collected. For reducing sugar determination, 0.1 mL supernatant was mixed with 0.1 mL 3,5-dinitrosalicylic acid (DNS) reagent, heated in a boiling water bath for 5 min, cooled to room temperature, and then diluted with 3 mL distilled water. Distilled water was used as the blank. Absorbance was measured at 540 nm, and the reducing sugar concentration was calculated using a glucose standard curve (Li, Fu, et al., 2025; Li et al., 2025).

#### 2.2.4 pH and electrical conductivity of L-Glu-starch suspensions

The pH and electrical conductivity of L-Glu-starch suspensions were directly measured at room temperature using a BPP-7800 laboratory precision pH meter and a DDSJ-308F conductivity meter.

#### 2.2.5 Scanning electron microscopy (SEM) observation of L-Glu-starch mixed samples

Samples were sputter-coated with gold using an ion sputter coater (Denton Vacuum, Moorestown, NJ, USA). Microstructures were observed using a field-emission scanning electron microscope (FE-SEM; SU8010, Hitachi, Japan) at an accelerating voltage of 5–10 kV (Zhang et al., 2018).

#### 2.2.6 Particle size distribution of L-Glu-starch mixed samples

Sample (0.2 g) was placed in a 2 mL centrifuge tube, mixed with 1.5 mL ultrapure water, and vortexed thoroughly. The starch granule suspension was then analyzed using a laser diffraction particle size analyzer (Microtrac S3500, Florida, USA) to obtain the volume-based particle size distribution (Zhang et al., 2018).

#### 2.2.7 Crystalline structure analysis of L-Glu-starch mixed samples

An appropriate amount of starch sample was evenly spread on a glass slide and analyzed using an X-ray diffractometer (Bruker D8 Advance, Germany). The scanning range was set to 2θ from 5 to 50 degrees with a scanning rate of 10 degrees/min (Govindaraju et al., 2022).

#### 2.2.8 Fourier transform infrared spectroscopy (FTIR) of L-Glu-starch mixed samples

An appropriate amount of sample was mixed with potassium bromide at a ratio of 1:100 (sample:KBr, w/w) and pressed into transparent pellets. FTIR spectra were recorded using an FTIR spectrometer (Thermo Fisher, USA) with a resolution of 4 cm^−1^, 32 scans, and a scanning range of 4000 to 400 cm^−1^. The intensity ratios of 1047/1022 and 1022/995 were used to evaluate the short-range order of starch (Wang et al., 2023).

#### 2.2.9 Raman spectroscopy of L-Glu-starch mixed samples

After calibrating the Raman spectrometer (Bruker), a small amount of sample was placed on a glass slide and flattened. Measurements were performed with an excitation wavelength of 785 nm, laser power set to 100%, and a scanning range of 2000 to 200 cm^−1^. The full width at half maximum (FWHM) at 480 cm^−1^ was calculated using a linear interpolation function (Li et al., 2025).

#### 2.2.10 Determination of solubility of glutamic acid-starch mixtures

Sample (200 mg) was mixed with 10 mL water to prepare a 2% suspension (*n* = 3). The suspension was heated in a water bath at 70 °C for 30 min, followed by cooling in an ice bath for 5 min. The mixture was centrifuged at 10,000 rpm for 20 min. The supernatant was dried at 105 °C to constant weight to determine the mass of solubilized starch (A). The calculations were performed as follows:

(1) $$\text{Solubility}\;\left(S,\%\right)=\left(A/200\right)\times 100\%$$

#### 2.2.11 Determination of swelling power of glutamic acid-starch mixture

Briefly, 200 mg of sample was accurately weighed into a pre-weighed 15 mL plastic centrifuge tube (A_1_), followed by the addition of 5 mL of deionized water. The mixture was thoroughly vortexed and heated in a water bath at 70 °C for 10 min with shaking, and then further heated in a boiling water bath for 10 min to ensure gelatinization. After heating, the samples were immediately cooled in cold water for 5 min and centrifuged at 1700×*g* for 4 min. The supernatant was carefully discarded, and the mass of the hydrated sediment together with the centrifuge tube was recorded (A_2_). Calculated as follows:

(2) $$\text{Swelling power}\;\left(g/g\right)=\left(A_{2}-A_{1}\right)/0.2\;$$

#### 2.2.12 Transparency of L-Glu-starch mixed samples

0.1 g sample was precisely weighed and diluted to 10 mL with distilled water to obtain a 1% suspension. The suspension was heated in a boiling water bath for 30 min. After cooling to 25 °C, absorbance was measured at 620 nm using distilled water as the blank control.

#### 2.2.13 Freeze-thaw stability of L-Glu-starch pastes/gels

A 6% sample paste was prepared, heated in a boiling water bath with stirring for 20 min, transferred to a centrifuge tube, and cooled to room temperature. The sample was stored at −20 °C for 24 h, then thawed naturally. After centrifugation at 10,000 rpm for 20 min at 25 °C, the supernatant was discarded. The precipitate was pressed with filter paper to remove surface water, and the precipitate mass was recorded to calculate syneresis.

(3) $$\text{Syneresis}\;\left(\%\right)=\left(m_{2}-m_{3}\right)/\left(m_{2}-m_{1}\right)\times 100\%$$

where m_1_ is the mass of the centrifuge tube (g); m_2_ is the total mass of the centrifuge tube plus starch paste before freezing (g); and m_3_ is the total mass of the centrifuge tube and the starch paste after thawing, centrifugation, pouring out the supernatant and absorbing the surface water (g).

#### 2.2.14 Differential scanning calorimetry (DSC) of L-Glu-starch samples

Sample (3 mg) was weighed into a specialized aluminum pan, and 9 μL deionized water was added. The pan was sealed and equilibrated at 4 °C for 12 h. Thermal properties were determined using a Q2000 differential scanning calorimeter (TA Instruments, USA). The thermograms were analyzed using the instrument software to obtain onset temperature (T_o_), peak temperature (T_p_), conclusion temperature (T_c_), and enthalpy change (ΔH).

#### 2.2.15 Pasting properties of L-Glu-flour samples (RVA, Rapid Visco Analyzer)

Pasting properties were measured using a Rapid Visco Analyzer (RVA 4500, Perten Instruments, Sweden). L-Glu powder was added to flour at 0% (w/w), 3.75% (w/w), and 7.5% (w/w) based on flour mass. The instrument software was used to obtain peak viscosity, trough viscosity, breakdown, final viscosity, setback, pasting time, and pasting temperature.

#### 2.2.16 SEM observation of L-Glu-starch gels

A 6% sample paste was heated in a boiling water bath with stirring for 30 min, transferred to centrifuge tubes, and cooled to room temperature. The samples were stored at 4 °C for 24 h to allow aging, then rapidly frozen in liquid nitrogen for 5 min and stored at −80 °C for 4 h. Samples were subsequently freeze-dried under vacuum for 48 h. Portions of the dried gels were cut and subjected to SEM observation following the same procedure as in Section 2.2.5.

#### 2.2.17 Structural characterization of L-Glu-starch gels

The freeze-dried samples from Section 2.2.16 were ground into powder and analyzed by XRD, FTIR, and Raman spectroscopy following the procedures described in Sections 2.2.7, 2.2.8, and 2.2.9, respectively.

### 2.3 Data processing

Microsoft Excel and SPSS 26.0 (IBM, New York, USA) were used for data organization and statistical analysis. Multiple comparisons were performed using Duncan's test, with significance set at *p* < 0.05. Figures were generated using Origin 2022.

## 3 Results

### 3.1 Effects of foliar L-Glu application on metabolite identification and abundance in grains under post-anthesis drought stress

**Fig. 1:**
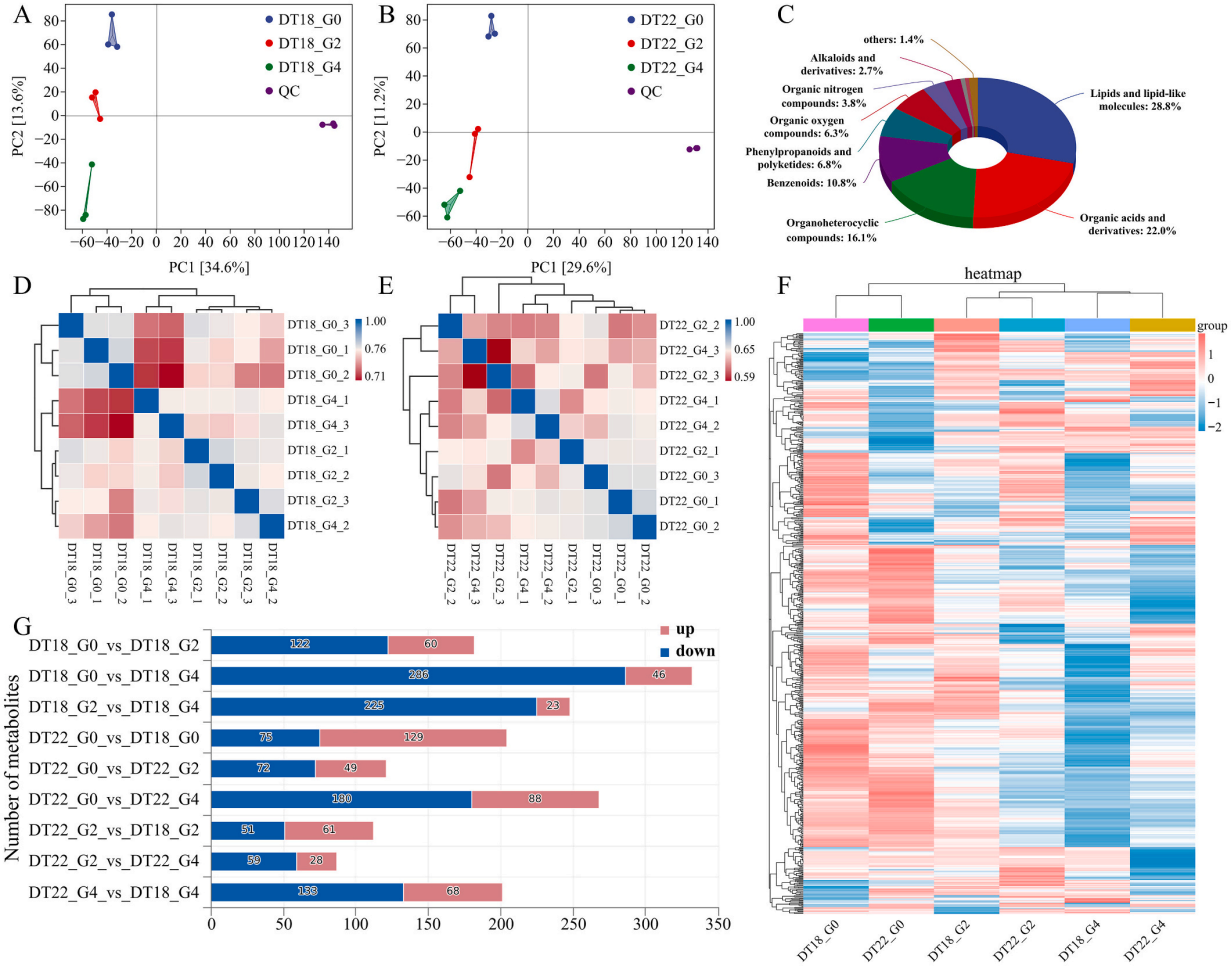
Effects of L-Glu application on grain metabolite identification and abundance under post-anthesis drought stress. DT18_G0: XD18 Drought Treatment +0 mmol/L L-Glu; DT18_G2: XD18 Drought Treatment +2 mmol/L L-Glu; DT18_G4: XD18 Drought Treatment +4 mmol/L L-Glu; DT22_G0: XD22 Drought Treatment +0 mmol/L L-Glu; DT22_G2: XD22 Drought Treatment +2 mmol/L L-Glu;DT22_G4: XD22 Drought Treatment +4 mmol/L L-Glu. A, B: the principal component analysis (PCA); C: the metabolite identification analysis; D, E: the sample correlation analysis; F: the metabolite clustering heatmap; G: the screening of differential metabolites.

As shown in Fig. 1A,B, PCA revealed a clear separation among samples from different treatments. The grain samples of 35 days post-anthesis exhibited relatively large inter-group distances and small intra-group distances. For the 35 d post-anthesis grain samples of XD18 and XD22, PC1 explained 34.6% and 29.6% of the total variance, respectively, whereas PC2 explained 13.6% and 11.2%, respectively. The cumulative explained variance reached 48.2% and 40.8%, respectively. Among all detected metabolites (POS), lipids and lipid-like molecules accounted for 28.8%, organic acids and derivatives for 22.0%, organoheterocyclic compounds for 16.1%, benzenoids for 10.8%, and phenylpropanoids and polyketides for 6.8% (Fig. 1C). Hierarchical clustering analysis was performed on all differential metabolites and visualized using a heatmap (Fig. 1F). Distinct regions of high or low abundance were observed for differential metabolites across treatment groups. Moreover, samples under the same L-Glu treatment exhibited strong inter-sample correlation, reflecting high experimental reproducibility (Fig. 1D,E). Quantification of differential metabolites across treatments further showed that, relative to DT_G0, the numbers of downregulated differential metabolites were higher than those of upregulated metabolites in both XD18 and XD22 under DT_G2 and DT_G4. In addition, compared with XD22, XD18 exhibited more upregulated than downregulated differential metabolites under DT_G0 and DT_G2 (Fig. 1G).

### 3.2 Effects of foliar L-Glu application on Venn analysis of differential grain metabolites and machine-learning-based feature selection under post-anthesis drought stress

**Fig. 2:**
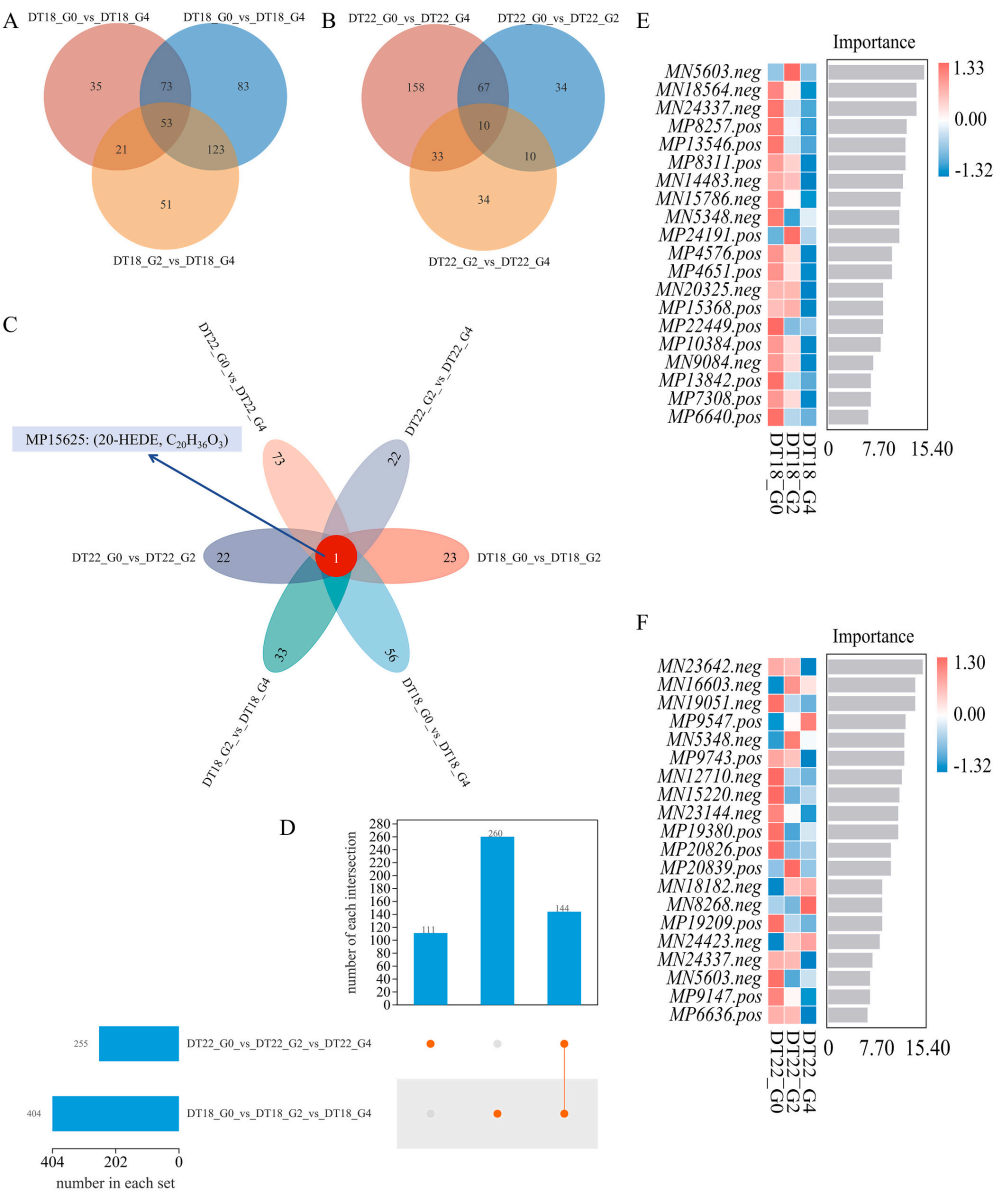
Venn analysis of differential metabolites and machine-learning-based analysis of differential metabolites under post-anthesis drought stress. DT18_G0: XD18 Drought Treatment +0 mmol/L L-Glu; DT18_G2: XD18 Drought Treatment +2 mmol/L L-Glu; DT18_G4: XD18 Drought Treatment +4 mmol/L L-Glu; DT22_G0: XD22 Drought Treatment +0 mmol/L L-Glu; DT22_G2: XD22 Drought Treatment +2 mmol/L L-Glu; DT22_G4: XD22 Drought Treatment +4 mmol/L L-Glu. A, B, C, D: Venn analysis of differential metabolites; E, F: Machine learning analysis of differential metabolites.

To quantify the numbers of treatment-specific and shared differential metabolites among different comparison groups, Venn analyses were conducted (Fig. 2A–C). The numbers of shared differential metabolites detected in the DT18_G0 vs DT18_G2, DT18_G0 vs DT18_G4, and DT18_G2 vs DT18_G4 comparisons were 53. In contrast, the numbers of shared differential metabolites detected in the DT22_G0 vs DT22_G2, DT22_G0 vs DT22_G4, and DT22_G2 vs DT22_G4 comparisons were 10. Notably, among these six comparison groups, only one differential metabolite was shared, namely 20-HEDE (MP15625, C_20_H_36_O_3_). The multi-group comparison DT18_G0 vs DT18_G2 vs DT18_G4 identified 404 differential metabolites, whereas DT22_G0 vs DT22_G2 vs DT22_G4 identified 255 differential metabolites; the two multi-group comparisons shared 144 differential metabolites (Fig. 2D).

Random forest machine-learning analysis of differential metabolites further indicated that, in the DT18_G0 vs DT18_G2 vs DT18_G4 comparison, the top five metabolites ranked by importance were: MN5603, m-cresol, 6-propyl; MN18564, 25-hydroxy-16,17,23,23,24,24-hexadehydrovitamin D3; MN24337, 6-O-((2E)-3-(4-Hydroxy-3-methoxyphenyl)prop-2-enoyl)hex-2-ulofuranosyl hexopyranoside; MP8257, L-Acetylcarnitine; and MP13546, 3-hydroxy-2-(3-nitro-4-piperidinobenzyl)propanenitrile. In the DT22_G0 vs DT22_G2 vs DT22_G4 comparison, the top five metabolites ranked by importance were: MN23642, N-{[(1*S*,4*S*,6*S*)-6-Isopropyl-3-methyl-4-{2-oxo-2-[4-(2-pyridinyl)-1-piperazinyl]ethyl}-2-cyclohexen-1-yl]methyl}-3-methoxypropanamide; MN16603, Moschamine; MN19051, Iguesterin; MP9547, 4,12,12-trimethyl-5-oxatricyclo[8.2.0.0,]dodecan-9-ol; and MN5348, l-Glutamine(Fig. 2E,F).

### 3.3 Effects of foliar L-Glu application on enrichment analysis and correlation analysis of differential grain metabolites under post-anthesis drought stress

**Fig. 3:**
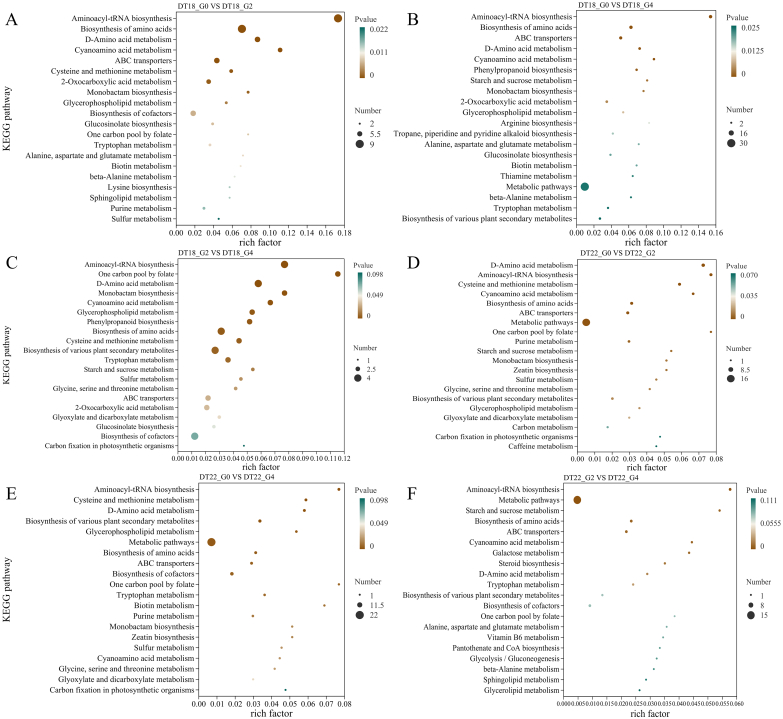
Enrichment analysis of differential metabolites in grains under post-anthesis drought stress. DT18_G0: XD18 Drought Treatment +0 mmol/L L-Glu; DT18_G2: XD18 Drought Treatment +2 mmol/L L-Glu; DT18_G4: XD18 Drought Treatment +4 mmol/L L-Glu; DT22_G0: XD22 Drought Treatment +0 mmol/L L-Glu; DT22_G2: XD22 Drought Treatment +2 mmol/L L-Glu; DT22_G4: XD22 Drought Treatment +4 mmol/L L-Glu.

To rapidly characterize the differences in differential metabolites among treatment groups and to infer potential functional tendencies of these metabolites, KEGG pathway enrichment analysis was conducted for the differential metabolites in each comparison (*p* < 0.05). The results are shown in Fig. 3. For the DT18_G0 vs DT18_G2 comparison, the main enriched pathways were aminoacyl-tRNA biosynthesis, cyanoamino acid metabolism, and D-Amino acid metabolism. For DT18_G0 vs DT18_G4, the main enriched pathways were aminoacyl-tRNA biosynthesis, cyanoamino acid metabolism, and arginine biosynthesis. For DT18_G2 vs DT18_G4, the main enriched pathways were one carbon pool by folate, aminoacyl-tRNA biosynthesis, and monobactam biosynthesis. For DT22_G0 vs DT22_G2, the main enriched pathways were aminoacyl-tRNA biosynthesis, one carbon pool by folate, and D-Amino acid metabolism. For DT22_G0 vs DT22_G4, the main enriched pathways were aminoacyl-tRNA biosynthesis, one carbon pool by folate, and biotin metabolism. For DT22_G2 vs DT22_G4, the main enriched pathways were aminoacyl-tRNA biosynthesis, starch and sucrose metabolism, and cyanoamino acid metabolism.

For the multi-group comparison DT18_G0 vs DT18_G2 vs DT18_G4, the major enriched pathways were Aminoacyl-tRNA biosynthesis, One carbon pool by folate, and Cyanoamino acid metabolism. For DT22_G0 vs DT22_G2 vs DT22_G4, the major enriched pathways were Aminoacyl-tRNA biosynthesis, Starch and sucrose metabolism, and One carbon pool by folate (Fig. S1). Pearson correlation analysis was performed using the top 30 differential metabolites. In both DT18_G0 vs DT18_G2 vs DT18_G4 and DT22_G0 vs DT22_G2 vs DT22_G4, a larger number of metabolites exhibited significant negative correlations. Specifically, MP15625 (20-HEDE), identified in Section 3.2, showed significant negative correlations with MP19209, MP14471, MP15500, MP20236, MP7390, MP15384, MP19146, MP15381, MP19257, MP23715, MP19551, MP24724, MP19500, MP36941, MP13724, MP24191, and other metabolites, respectively, while showing significant positive correlations with MP3941, MP5023, MP2347, MP18675, and other metabolites, respectively (Fig. S1; Table S5).

### 3.4 Effects of L-Glu addition on enzymatic hydrolysis of starch granules under post-anthesis drought stress

Observations by confocal laser scanning microscopy (CLSM) showed that, in both wheat cultivars, starch granules from the DT_G0 treatment displayed extensive, strong, and distinct fluorescence. The fluorescent dye entered the interior of starch granules through micropores and microchannels. Therefore, starch from the DT_G0 treatment was selected as native starch for modification. L-Glu was added directly to this starch, followed by α-amylase hydrolysis. The concentration of reducing sugars released was determined, and the results indicated that starch with directly added L-Glu produced a higher concentration of reducing sugars (Fig. S2 E,F).

### 3.5 Effects of L-Glu addition on the physicochemical properties of starch suspensions and on granule morphology and particle-size distribution

As shown in Table S1, the pH values of starch samples in the control group (without L-Glu addition, 0%) were 7.21 and 7.10, respectively. With increasing L-Glu addition, the acidity and electrical conductivity of the system increased progressively. When the L-Glu addition reached 7.5%, the pH values of the L-Glu–starch blended systems decreased to 3.59 and 3.47, respectively, indicating an acidic environment (Table S1).

**Fig. 4:**
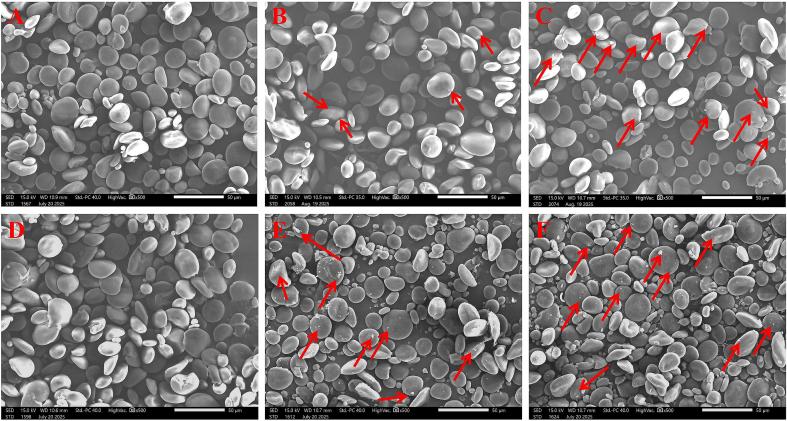
Effect of L-Glu addition on starch granule morphology. A, D: starch from DT18_G0 and DT22_G0 with 0% L-Glu added, respectively; B, E: starch from DT18_G0 and DT22_G0 with 3.75% L-Glu added, respectively; C, F: starch from DT18_G0 and DT22_G0 with 7.5% L-Glu added, respectively. The red arrows indicate white particles adhering to the surface of the starch granules. (For interpretation of the references to colour in this figure legend, the reader is referred to the web version of this article.)

As shown in Fig. 4, native wheat starch granules were irregular in size and exhibited round or oval shapes, with particle sizes ranging from 0 to 40 μm. Different L-Glu addition levels (0–7.5%, w/w) had no significant effect on the surface morphology of starch granules; however, a certain number of white particles were observed adhering to the granule surfaces, and more such particles were attached to the surface of XD22 starch (Fig. 4E,F). Particle-size distribution measurements further showed that different L-Glu addition levels (3.75–7.5%) significantly increased both the mean volume diameter and the surface area–weighted mean diameter of starch (Table 1).

**Table 1:** Effect of L-Glu addition on starch particle size distribution.

Starch	Glu contents/%	Particle size parameters (μm)		
		Volume-weighted mean particle diameter (volume)	Number-weighted mean particle diameter (diameter)	Surface area–weighted mean particle diameter (specific surface area)
DT18_G0	0	21.30 ± 0.05d	6.41 ± 0.01c	17.64 ± 0.01e
	3.75	25.97 ± 0.45a	13.98 ± 0.46a	21.65 ± 0.33a
	7.5	25.13 ± 0.17b	13.68 ± 0.02a	21.36 ± 0.10a

DT22_G0	0	21.74 ± 0.01d	7.75 ± 0.00b	18.36 ± 0.01d
	3.75	23.59 ± 0.18c	7.63 ± 0.02b	19.09 ± 0.13c
	7.5	24.32 ± 0.26c	7.73 ± 0.03b	19.86 ± 0.17b

Note: The data presented in the table are expressed as mean ± standard error. Different letters indicate a significant difference at the *p* < 0.05 level.

### 3.6 Effects of L-Glu addition on the crystalline structure, chemical bonds, and functional groups of starch samples

**Fig. 5:**
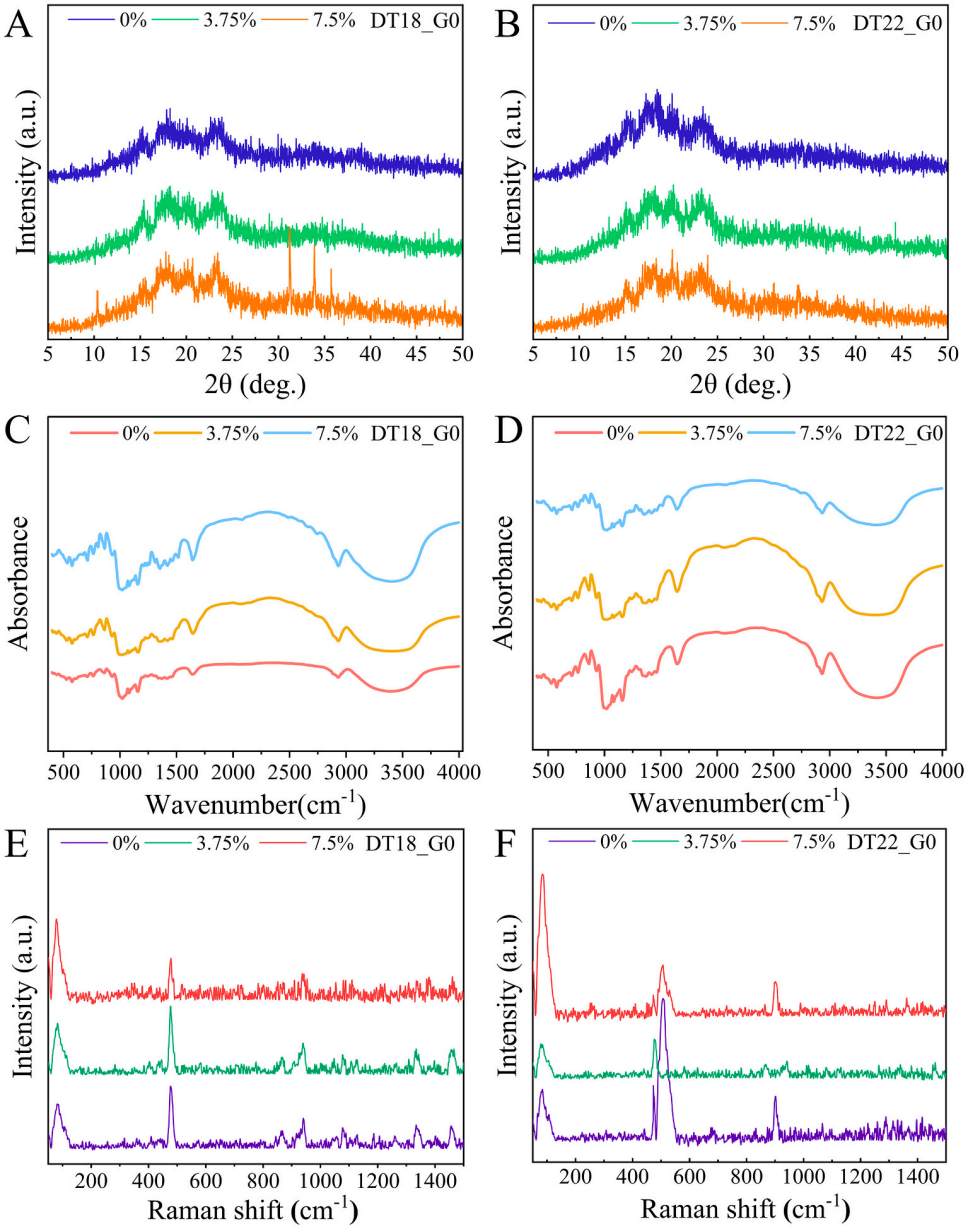
Effects of L-Glu addition on starch characterization. A, B: X-ray diffraction (XRD) analysis; C, D: Fourier transform infrared (FTIR) spectroscopy; E, F: Raman spectroscopy.

X-ray diffraction (XRD) was used to examine the crystalline structure and modification of starch. Distinct diffraction peaks were observed for the L-Glu–starch samples at 2θ = 15°, 17°, 18°, and 23°, and a connected doublet peak was observed near 2θ = 17° and 18°, which is characteristic of the typical A-type pattern of cereal starch. After adding different amounts of amino acids, no obvious changes were observed in the A-type characteristic diffraction peaks of wheat starch (Fig. 5A,B). The short-range ordered structure of L-Glu–starch samples was assessed by FTIR. As shown in Fig. 5, a broad absorption band was observed in the region of 3000–3700 cm^−1^. After L-Glu addition, this band became broader, but no new absorption peaks appeared (Fig. 5C,D). In addition, after L-Glu addition, the 1047/1022 and 1022/995 ratios of the mixed samples exhibited a trend of first increasing and then decreasing, whereas the 1022/995 ratio of the DT22_G0 starch sample showed a decreasing trend (Table S2). Raman spectra of starch typically exhibit vibrational features in the 400–800 cm^−1^ region arising from C–C–O and C–C–C deformation. In the present study, the FWHM at 480 cm^−1^ increased after L-Glu addition (Table S3). Moreover, the intensity of the characteristic peak at 480 cm^−1^ decreased significantly with increasing L-Glu concentration (Fig. 5E,F).

### 3.7 Effects of L-Glu addition on the physicochemical properties of starch samples

The swelling power, transparency, solubility, and freeze–thaw stability of wheat starch with L-Glu addition are shown in Fig. S3. Negatively charged L-Glu significantly reduced the swelling power and transparency of wheat starch, and both parameters decreased progressively with increasing L-Glu concentration. Notably, L-Glu significantly increased the solubility and syneresis (water separation rate) of wheat starch, resulting in poorer freeze–thaw stability (Fig. S3).

### 3.8 Effects of L-Glu addition on gelatinization and phase transition of starch samples

Differential scanning calorimetry (DSC) was used to elucidate how L-Glu addition regulates the thermodynamic properties of starch. The results showed that, after adding different concentrations of L-Glu, the onset temperature (T_o_), peak temperature (T_p_), and conclusion temperature (T_c_) all increased to varying extents. For DT22_G0 starch, 3.75% L-Glu also led to a significant increase in enthalpy (ΔH), whereas other treatments showed no consistent change in ΔH (Table 2).

**Table 2:** Influence of L-Glu addition on thermodynamic parameters of starch.

Starch	Glu contents/%	T_o_ (°C)	T_p_ (°C)	T_c_ (°C)	△H (J/g)
DT18_G0	0	63.59 ± 0.06c	66.29 ± 0.06b	69.14 ± 0.08ab	6.64 ± 0.25bc
	3.75	63.78 ± 0.05bc	66.30 ± 0.06b	69.13 ± 0.04ab	8.40 ± 0.30ab
	7.5	64.30 ± 0.21a	66.76 ± 0.12a	69.52 ± 0.20a	7.33 ± 0.65abc

DT22_G0	0	62.99 ± 0.05d	65.46 ± 0.11c	68.19 ± 0.15c	6.34 ± 0.25c
	3.75	63.95 ± 0.01b	66.37 ± 0.06b	69.08 ± 0.03b	8.93 ± 0.25a
	7.5	64.61 ± 0.12a	66.79 ± 0.13a	69.37 ± 0.09ab	6.76 ± 1.21bc

Notes: DT18_G0: XD18 Drought Treatment +0 mmol/L L-Glu (field); DT22_G0: XD22 Drought Treatment +0 mmol/L L-Glu (field); The data presented in the table are expressed as mean ± standard error. Different letters indicate a significant difference at the *p* < 0.05 level.

The viscosity changes of wheat flour during the heating–cooling process were further examined to evaluate the effect of different L-Glu concentrations on RVA properties. The results indicated that L-Glu addition altered the RVA characteristics of wheat flour. Specifically, adding 3.75% L-Glu significantly increased the breakdown viscosity of DT18_G0 flour and significantly decreased the final viscosity and setback viscosity. Adding 7.5% L-Glu significantly increased the pasting temperature of DT18_G0 flour. Moreover, adding 7.5% L-Glu significantly increased the breakdown viscosity of DT22_G0 flour and significantly decreased the final viscosity (Table 3). Overall, these results indicate that the effects of L-Glu on RVA pasting parameters were cultivar- and concentration-dependent.

**Table 3:** Influence of L-Glu addition on wheat flour gelatinization parameters.

Starch	Glu contents/%	Breakdown viscosity (cP)	Final viscosity (cP)	Setback viscosity (cP)	Pasting time (min)	Pasting temperature (°C)
DT18_G0	0	355.33 ± 11.79b	3394.67 ± 31.16a	1539.00 ± 44.09a	5.65 ± 0.02ab	79.17 ± 2.01c
	3.75	622.67 ± 39.01a	3022.67 ± 5.33bc	1300.00 ± 14.57b	5.53 ± 0.00b	84.60 ± 0.20ab
	7.5	339.67 ± 40.81b	2873.67 ± 11.78d	1056.00 ± 44.54c	5.53 ± 0.04b	88.85 ± 0.23a

DT22_G0	0	308.67 ± 62.14b	3120.67 ± 67.68b	1285.33 ± 116.01b	6.13 ± 0.37a	82.22 ± 0.42bc
	3.75	405.33 ± 33.51b	3051.67 ± 63.57b	1282.33 ± 17.57b	5.49 ± 0.06b	83.53 ± 1.66bc
	7.5	570.00 ± 12.74a	2910.33 ± 30.47d	1185.67 ± 17.65bc	5.62 ± 0.02ab	85.67 ± 2.21ab

Notes: DT18_G0: XD18 Drought Treatment +0 mmol/L L-Glu (field); DT22_G0: XD22 Drought Treatment +0 mmol/L L-Glu (field); The data presented in the table are expressed as mean ± standard error. The mean values in the table are calculated from three replicates. Different letters indicate a significant difference at the *p* < 0.05 level.

### 3.9 Concentration-dependent effects of L-Glu on starch structural stability and conformation

**Fig. 6:**
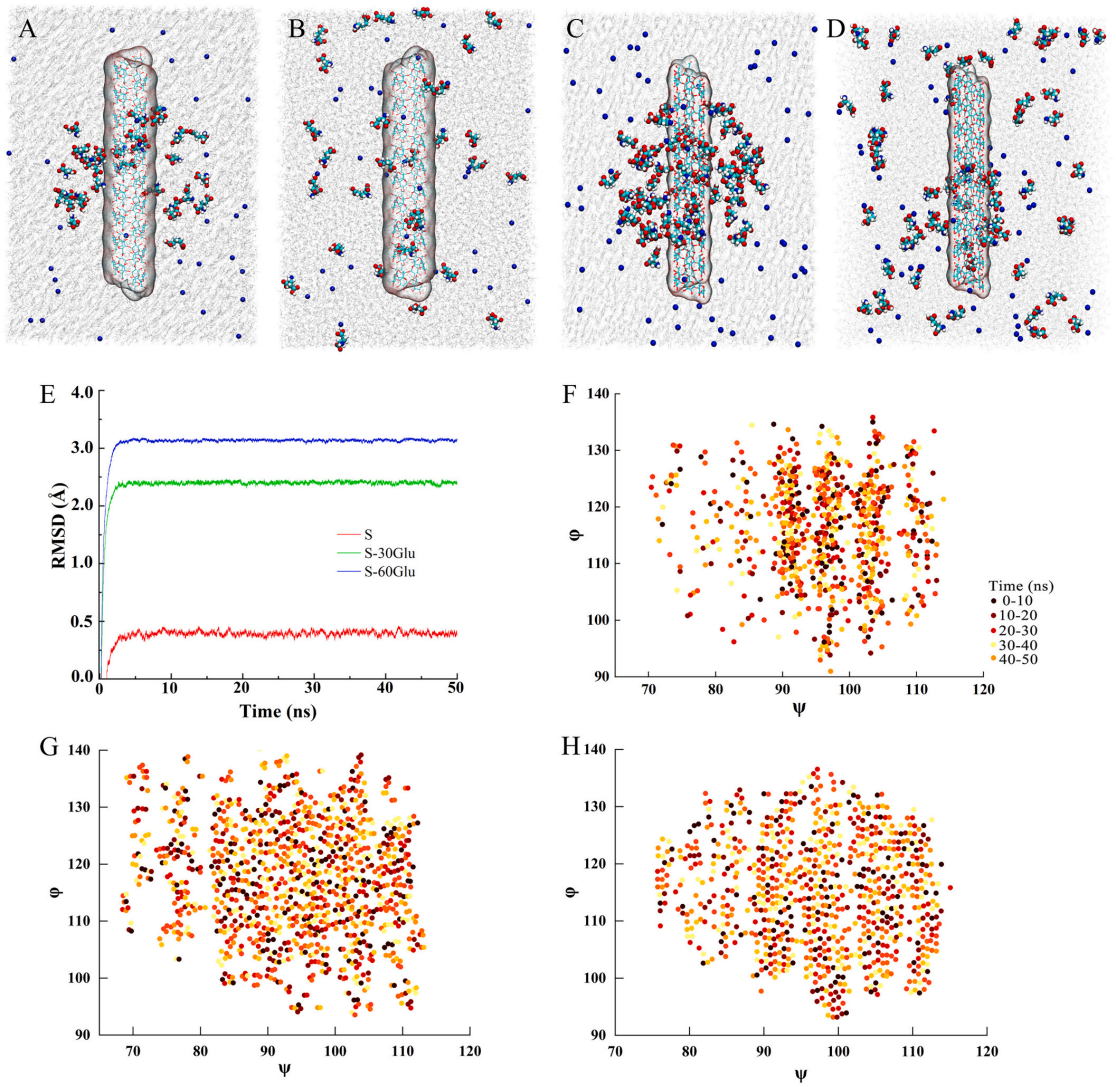
Effects of L-Glu on the concentration-dependent changes in starch structural stability and conformation. A–D show molecular dynamics simulation snapshots of systems with different glutamic acid concentrations. Snapshots of the low-concentration glutamic acid system (S-30Glu) at 0 ns (A) and 50 ns (B), and snapshots of the high-concentration glutamic acid system (S-60Glu) at 0 ns (C) and 50 ns (D). In panels A–D, the linear structures represent starch chains; oxygen, carbon, nitrogen, and hydrogen atoms are shown in red, cyan, blue, and white, respectively; the free blue spheres represent sodium ions (Na^+^). Panel E shows the root-mean-square deviation (RMSD) analysis. Panels F–H show dihedral angle distributions of starch chains in the starch–water system (F), the starch–low-concentration glutamic acid system (G), and the starch–high-concentration glutamic acid system (H). (For interpretation of the references to colour in this figure legend, the reader is referred to the web version of this article.)

Molecular dynamics simulations indicated that all simulated systems reached conformational equilibrium at the early stage of the production runs (<10 ns), and the RMSD of starch chains converged to a plateau, demonstrating good system stability. Simulation snapshots (Fig. 6A–D) showed that water molecules promoted solvation by forming hydrogen bonds with starch chains. L-Glu molecules were initially randomly distributed around starch and gradually became more evenly dispersed in the aqueous phase as the simulation proceeded. RMSD analysis further revealed a pronounced concentration-dependent trend in conformational stability of starch chains: pure-water system (S) < low-concentration glutamate system (S-30Glu) < high-concentration glutamate system (S-60Glu) (Fig. 6E). This indicates that higher L-Glu concentrations lead to greater fluctuations and lower stability of the starch single-helix structure.

The distributions of key dihedral angles of the α-1,4-glycosidic bond (ϕ/O5–C1–O4′–C4′ and ψ/C1–O4′–C4′–C3′) further corroborated this pattern (Fig. 6F–H; Fig. S5). In the pure-water starch system, the ϕ angle (90°–135°) and ψ angle (70°–112°) exhibited concentrated distributions, indicating a regular helical conformation. After introducing L-Glu, the dihedral-angle distributions expanded markedly: in the low-L-Glu group, the ϕ angle broadened to 90°–140°, whereas in the high-L-Glu group, the ψ angle further extended to 75°–115°. These changes directly reflect increased conformational flexibility of glycosidic bonds, disruption of the short-range ordered structure and helical regularity of starch, and an enhancement of the perturbation effect with increasing L-Glu concentration.

### 3.10 Intermolecular interactions between L-Glu and starch molecules and spatial distribution characteristics

Hydrogen bond and interaction energy analyses (Table 4) demonstrated that the introduction of L-Glu did not substantially disrupt the internal hydrogen bond network within amylose, with the number of starch-starch hydrogen bonds remaining stable at 144–146. However, it markedly reshaped the hydrogen-bond distribution of the system. L-Glu formed 1.47–2.18 (on average) stable intermolecular hydrogen bonds with starch chains per system (total interaction energy, −169.3 to −205.3 kJ/mol), while simultaneously forming a large number of hydrogen bonds with water (305.9–605.3), thereby competitively occupying hydration sites on the starch surface. Consequently, the number of starch–water hydrogen bonds slightly decreased from 129.5 to 126.1 (Table 4).

**Table 4:** Number of hydrogen bonds and interaction energies between different molecules in various systems.

Treatment	Hydrogen bonds numbers				Interaction energy/(kJ/mol)	
	S-S	S-W	S-G	G-W	S-W	S-G
S	146	129.5	/	/	−4726.2	/
S-30Glu	144.5	126.3	1.47	305.9	−4518.6	−169.3
S-60Glu	144.6	126.1	2.18	605.3	−4518.1	−205.3

Note: S-S: starch-starch; S-W: starch-water; S-G: starch- glutamic acid; G-W: glutamic acid-water. The interaction energy is the sum of short-range electrostatic interaction energy and short-range van der Waals interaction energy.

**Fig. 7:**
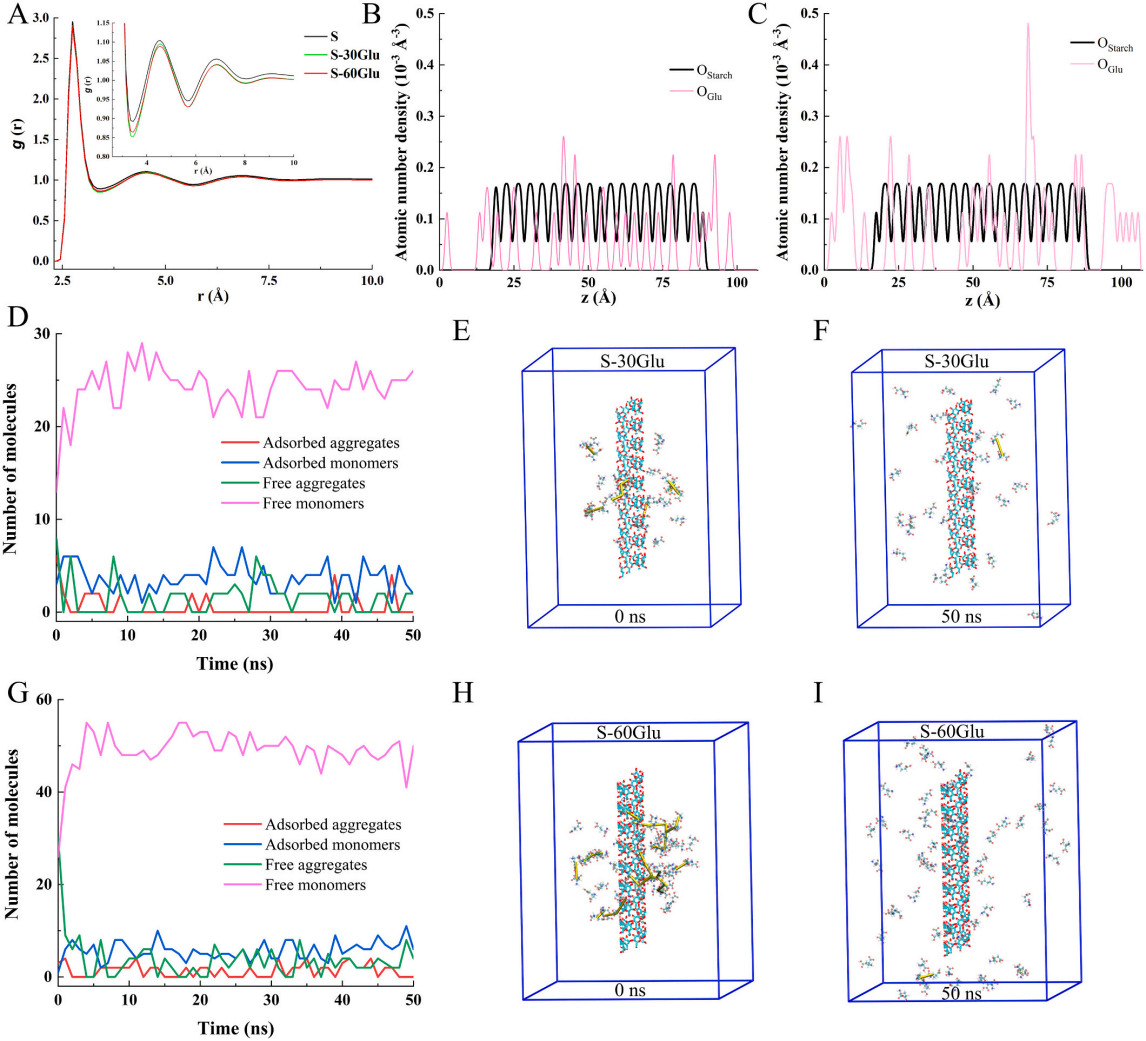
Investigation of the intermolecular interactions between L-Glu and starch and their spatial distribution characteristics. A shows the radial distribution functions (RDFs) of different simulated systems. B and C show the atomic density distributions along the z-axis for the S-30Glu (B) and S-60Glu (C) systems, respectively. D and G present analyses of glutamic acid in aggregated or monomeric states in the S-30Glu (D) and S-60Glu (G) systems: the red line (Adsorbed aggregates) indicates the number of glutamic acid aggregates adsorbed on the starch surface (i.e., aggregates formed by multiple glutamic acid molecules that subsequently bind to starch); the blue line (Adsorbed monomers) indicates the number of glutamic acid monomers adsorbed on the starch surface as single molecules; the green line (Free aggregates) indicates the number of glutamic acid aggregates not adsorbed on starch and free in the aqueous phase; and the pink line (Free monomers) indicates the number of glutamic acid monomers not adsorbed on starch and free in the aqueous phase. E, F, H, and I show simulation snapshots of glutamic acid aggregation behavior in the S-30Glu and S-60Glu systems. Snapshots of the S-30Glu system at 0 ns (E) and 50 ns (F) and of the S-60Glu system at 0 ns (H) and 50 ns (I) illustrate the distribution of glutamic acid in aggregated or free states. The yellow short sticks in the figure indicate the connections between the centers of mass of glutamic acid molecules within the same aggregate, which are used to characterize the aggregation state of glutamic acid. (For interpretation of the references to colour in this figure legend, the reader is referred to the web version of this article.)

Radial distribution function (RDF) analysis further showed (Fig. 7A) that all systems exhibited a characteristic O–H···O peak at 2.8 Å, corresponding to the hydration layer of starch–water hydrogen bonding. After adding L-Glu, the overall g(r) values decreased, and the g(r) values in the fluctuation region further declined in the high-concentration group, indicating weakened stability of the hydration layer around starch. This phenomenon was attributed to hydrogen-bond competition by L-Glu and steric hindrance effects introduced by its charged groups.

*Z*-axis atomic density distribution analysis revealed a concentration-dependent interaction between glutamate and starch (Fig. 7B,C). At low concentration, the oxygen-atom density of starch displayed periodic fluctuations within the 20–90 Å range, forming an ordered layered structure. The oxygen-atom density of glutamate was low, and its peak positions coincided with those of starch, indicating selective adsorption on the starch surface or between layers without disrupting long-range order. At high concentration, glutamate density fluctuations intensified and peak values increased; the correlation with starch density peaks weakened, suggesting that a large amount of glutamate inserted between starch chains and interfered with the ordered structural network.

The number-evolution curves and simulation snapshots indicated that the existence state of glutamate exhibited a clear concentration dependence (Fig. 7D–I). In the S-30Glu (low-concentration) system, free monomers were dominant (stabilizing at approximately 25), whereas the numbers of “adsorbed monomers” and “adsorbed aggregates” remained at low levels (<10). In the S-60Glu (high-concentration) system, the number of free monomers increased to more than 50, while the two types of aggregates remained at low levels (1–2). Meanwhile, although the numbers of “adsorbed monomers” and “free aggregates” increased, their proportions relative to the total number of molecules did not rise significantly, indicating that aggregation behavior did not intensify with increasing concentration. Snapshots further visually demonstrated that glutamate existed in a dispersed monomeric form in both systems, without local enrichment or dense aggregation.

### 3.11 Effects of L-Glu addition on the microstructure and structural characterization of starch gels

**Fig. 8:**
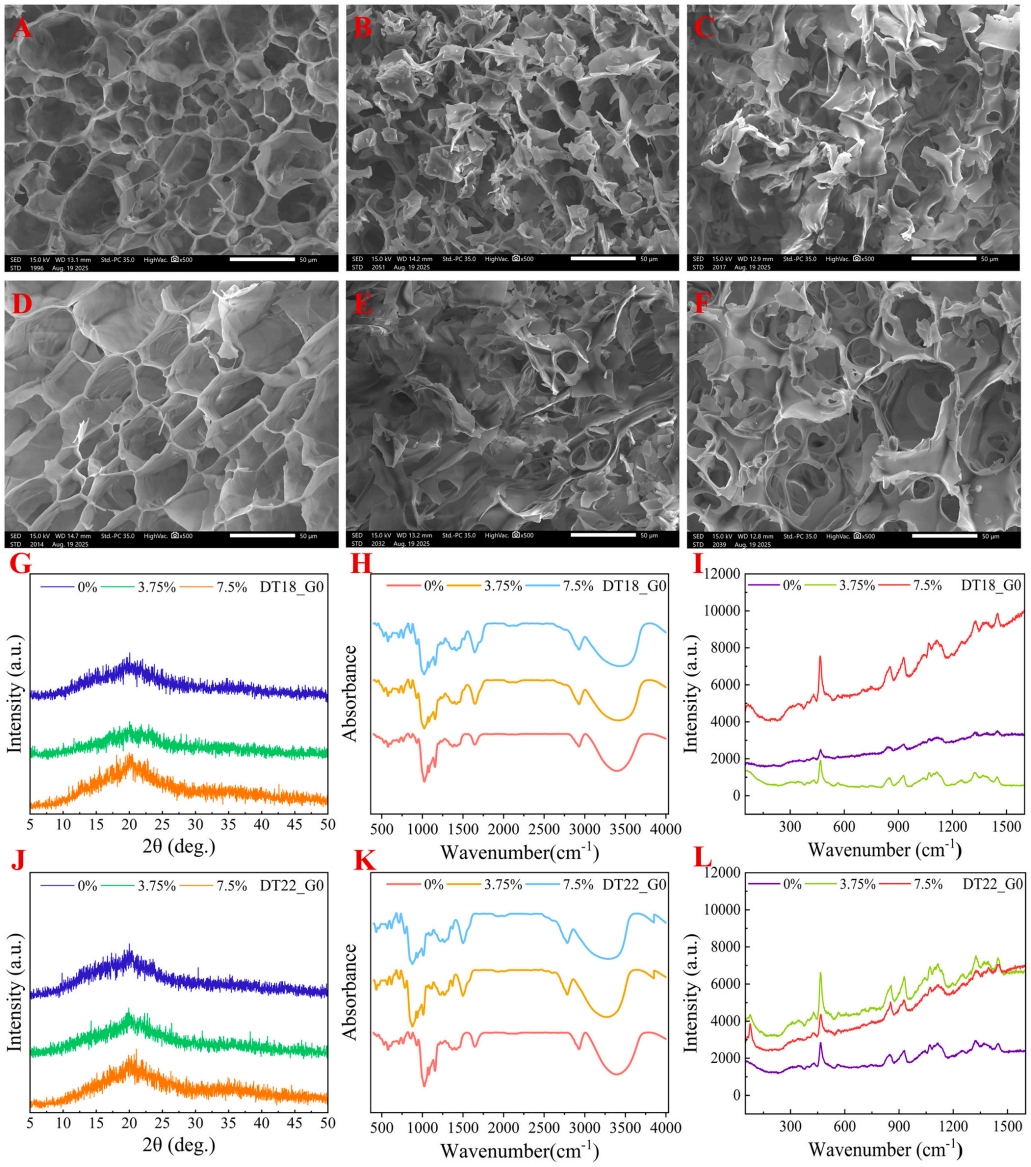
Effects of L-Glu addition on the microstructure and characterization of starch gels. A, D: starch gels prepared from DT18_G0 and DT22_G0 with 0% L-Glu added, respectively; B, E: starch gels prepared from DT18_G0 and DT22_G0 with 3.75% L-Glu added, respectively; C, F: starch gels prepared from DT18_G0 and DT22_G0 with 7.5% L-Glu added, respectively; G, J: X-ray diffraction (XRD) analysis; H, K: Fourier transform infrared (FTIR) spectroscopy; I, L: Raman spectroscopy.

SEM observations of starch gels showed that gels formed by wheat starch without L-Glu exhibited an interlaced, grid-like crosslinked surface structure with numerous internal pores (Fig. 8A,D). In contrast, after adding different concentrations of L-Glu, the gel structures of the mixed samples differed markedly from those of the native starch gel. The gels failed to form a uniform and well-defined porous structure; instead, the microstructure appeared lamellar, with many fine fragmented block-like structures, and the overall structure was unevenly distributed (Fig. 8B,C,E,F).

The XRD pattern of starch hydrogels showed a broad amorphous halo at 19.5–20°, indicating that gelatinization destroyed the original A-type crystalline structure. After retrogradation, the starch chains remained predominantly disordered, which differed clearly from the native A-type pattern of cereal starch. Compared with the native starch gel, the O—H stretching vibration band near 3400 cm^−1^ in L-Glu-added wheat starch gels became broader and shifted, whereas the other absorption peaks were very close. In addition, the stretching vibration peaks associated with C—H bonds (near 2940 cm^−1^) and fingerprint-region vibrations such as C—C, C—N, and C—O (near 1330–667 cm^−1^) also showed shifts to certain extents. Raman spectra of the gels indicated that starch gels exhibited a feature of “hindered development of order,” which was markedly different from the Raman spectra of starch before gelatinization (Fig. 8I,L). Furthermore, adding 3.75% L-Glu decreased the intensity ratio I_1122_/I_1096_ (at 1122 cm^−1^ and 1096 cm^−1^), whereas adding 7.5% L-Glu increased I_1122_/I_1096_ (Table S4).

## 4 Discussion

### 4.1 Foliar application of L-Glu reprograms grain metabolism to optimize the metabolic background for starch biosynthesis under post-anthesis drought stress

During abiotic stress and the concomitant deterioration of crop quality, plant metabolites can change rapidly in response to stress (Hou et al., 2025). In this study, untargeted metabolomics revealed that exogenous foliar application of L-Glu significantly reshaped the metabolic profile of wheat grains under post-anthesis drought stress. PCA showed clear separation among treatments, confirming that both post-anthesis drought stress and L-Glu treatment can induce pronounced physiological and metabolic reorganization. Notably, KEGG enrichment analysis revealed that the differential metabolites were significantly enriched in key pathways including Aminoacyl-tRNA biosynthesis, Starch and sucrose metabolism, and Cyanoamino acid metabolism (Fig. 3). This finding not only demonstrates that exogenous L-Glu can be incorporated into the primary metabolic network of wheat, but more importantly, the metabolomic data in this study mutually corroborate the physiological conclusions of our previously published research regarding the regulation of starch synthesis by L-Glu (Li et al., 2025), providing correlative evidence and research directions for dissecting the upstream metabolic mechanism underlying this regulatory effect.

This study further elucidates how such metabolic reorganization creates a favorable intracellular environment for starch synthesis. First, foliar L-Glu application induced differential accumulation of a large number of primary metabolites, such as lipids and organic acids (Fig. 1), suggesting that L-Glu may act as a key metabolic node by modulating the allocation of carbon skeletons through the tricarboxylic acid (TCA) cycle and energy (ATP) supply, thereby ensuring the precursors and energy required for starch biosynthesis (Li et al., 2024; Liao et al., 2022). Second, enrichment of the “Aminoacyl-tRNA biosynthesis” pathway suggests that L-Glu treatment may enhance translational capacity for protein synthesis. In addition, the key differential metabolite identified by differential metabolite venn analysis—oxylipin 20-HEDE (Fig. 2)—showed L-Glu-regulated abundance, further indicating that L-Glu may go beyond its role as a substrate and fine-tune grain carbon-allocation strategies by modulating stress-signaling pathways. This cross-scale regulation—from “stress signal perception” to “metabolic profile control”—systematically optimizes the “metabolic background” for starch synthesis.

Taken together, exogenous foliar L-Glu application acts as a metabolic regulator that systematically reprograms the grain metabolic network under drought stress. Such reprogramming is not random; rather, by enhancing precursor supply, safeguarding the potential of biosynthetic enzymes, and fine-tuning carbon-allocation signals, it synergistically creates a more favorable intracellular environment for starch biosynthesis and high-quality accumulation under stress conditions (Li et al., 2025). These results provide a solid physiological basis for understanding how L-Glu indirectly alleviates drought-induced deterioration of grain quality via physiological and metabolic pathways, and they form a clear mechanistic contrast with the subsequent in vitro effects where L-Glu directly functions as a “structural regulator”.

### 4.2 L-Glu directly regulates starch structure and functional properties across multiple scales via intermolecular interactions

Previous studies have shown that amino acids can actively and multi-scalely alter the aggregated structure of starch through intermolecular interactions, thereby determining its final functionality (Xu et al., 2022). In the present study, FTIR and Raman results jointly showed that L-Glu addition broadened the O—H stretching vibration band (near 3400 cm^−1^) and increased the Raman full width at half maximum (FWHM) at 480 cm^−1^ (Fig. 5; Table S3). These observations indicate that the carboxyl and amino groups of L-Glu form new hydrogen bonds with starch hydroxyl groups, competitively disrupting the originally ordered hydrogen-bond network between starch molecular chains and increasing disorder in the double-helix structure (Xu et al., 2022). Moreover, the absence of new absorption peaks suggests that the two components are associated via non-covalent interactions, i.e., no covalent interaction exists between the amino acid and starch (Xu et al., 2023). Meanwhile, the ratios 1047/1022 and 1022/995 in the mixed samples changed to varying extents after L-Glu addition, indicating a “concentration effect” of L-Glu action. High glutamate concentrations may lead to excessive insertion and/or competitive hydrogen bonding, disrupting starch chain order, increasing the amorphous region, and reducing the degree of short-range order (Fig. 5; Table S2).

Molecular dynamics simulations further validated the above mechanism at the atomic level. L-Glu formed 1.47–2.18 stable intermolecular hydrogen bonds with starch chains per system, with a total interaction energy of −169.3 to −205.3 kJ/mol per system (Table 4). This binding exhibited a concentration-dependent effect: as L-Glu concentration increased, the RMSD of the starch single helix increased significantly, and the distribution ranges of the α-1,4-glycosidic bond dihedral angles (ϕ, ψ) broadened, directly reflecting enhanced flexibility and reduced regularity of the starch helical conformation (Fig. 6). Meanwhile, radial distribution function (RDF) analysis showed that L-Glu has a stronger hydrogen-bonding capacity with water molecules (the high-concentration group formed 605.3 G–W hydrogen bonds), thereby competitively occupying hydration sites on the starch surface. Consequently, the number of starch–water (S—W) hydrogen bonds decreased from 129.5 to 126.1, disrupting the stable hydration layer around starch (Table 4; Fig. 7). Under the acidic conditions of the blend system (pH 3.5–3.6), the carboxyl groups of L-Glu are partially dissociated and carry a weak negative charge; the electrostatic repulsion and steric hindrance generated upon adsorption may further impede the approach and ordered packing of starch chains. This is consistent with the molecular dynamics observation that, at both low and high concentrations, L-Glu predominantly exists as free monomers without obvious aggregation (Fig. 7).

This molecular-level disordering is corroborated at higher structural and functional levels. XRD showed that the A-type crystalline polymorph of starch was not altered after L-Glu addition; however, the weakened Raman characteristic peaks and increased FWHM collectively indicate that L-Glu insertion disrupts short-range order in starch, functioning as a “molecular-level” physical modification. This “loosening” of structure directly leads to functional changes. Hydrogen bonds formed between L-Glu and starch disrupt the original molecular network; together with the simulation-demonstrated decrease in the stability of the starch hydration layer, starch granules become less able to effectively retain water, resulting in a significant decrease in swelling power. Meanwhile, amylose in the amorphous region may dissolve more rapidly (Ito, Hattori, Yoshida, Watanabe, et al., 2006; Komiya & Nara, 1986), further weakening the integrity of starch granule structure. In addition, CLSM results showed that drought stress (DT_G0) itself generated more micropores and microchannels in starch granules (Fig. S2), which to some extent caused abnormal development of crystalline and amorphous regions in starch granules (Li et al., 2025). The adhesion of L-Glu further renders the granule surface “looser and more disordered”, providing more action sites for α-amylase and thereby significantly increasing the in vitro digestion rate, consistent with the findings of Yue et al. (2022).

L-Glu addition also significantly affected the thermodynamic properties of starch and the pasting properties of wheat flour. DSC results showed that T_o_, T_p_, and T_c_ increased to varying extents, indicating that L-Glu exerts a “protective effect” on starch granule structure through molecular interactions and enhances thermal stability (Ito, Hattori, Yoshida, & Takahashi, 2006; Liang & King, 2003). Nevertheless, such temperature elevation is mainly attributed to L-Glu competing for hydration water and restricting granule swelling, rather than enhanced intrinsic crystalline order of starch. RVA analysis further showed that L-Glu increased breakdown viscosity but significantly decreased final viscosity and setback viscosity.

Xu et al. (2022) reported that amino acids can increase starch thermal parameters, which primarily arise from interactions between amino and carboxyl groups and starch hydroxyl groups, or weak interactions between amino acid side chains and starch hydroxyl groups that protect starch granule structure (Yue et al., 2022). This is consistent with the simulation results in the present study showing “stable hydrogen-bond binding between L-Glu and starch”, which together enhances starch granule structural stability and thus increases the onset gelatinization temperature. The decrease in setback viscosity caused by L-Glu indicates that L-Glu can inhibit short-term starch retrogradation, mainly due to the interactions between L-Glu and starch molecules and the acidity of the L-Glu solution. On the other hand, after gelatinization, the starch crystalline structure becomes loosened and steric hindrance decreases, which facilitates L-Glu binding and occupation of hydrogen-bond sites, thereby preventing association and rearrangement of starch chains (Yue et al., 2022). Meanwhile, simulations showed that L-Glu is mainly dispersed as free monomers in solution, exerting a dilution effect on starch paste, which may ultimately lead to increased breakdown viscosity and decreased setback viscosity.

### 4.3 A dual regulatory mechanism of L-Glu on starch gel network formation and aging behavior

The properties of starch gels depend on the rearrangement and retrogradation of molecular chains after gelatinization. In this study, L-Glu addition exerted a dual regulatory effect on gel network formation, in which “disruption” and “reconstruction” coexisted. SEM images clearly showed that gels with L-Glu addition did not form a uniform, porous three-dimensional network; instead, disordered lamellar and fragmented block-like structures were observed (Fig. 8). This may mainly be attributed to L-Glu–induced disruption of the inherent granule structure before gelatinization, which reduces the number of intact molecular chains available for crosslinking after gelatinization and thereby weakens the network skeleton. Moreover, the polar groups of L-Glu confer hydrophilicity, which may alter the interactions between starch and water (Xu et al., 2025). In addition, during the cooling and aging stage, L-Glu molecules occupy rearrangement sites of amylose and amylopectin side chains through steric hindrance and competition for hydrogen bonding with water, thereby significantly inhibiting the formation of double-helix crosslinking junctions.

With increasing concentrations of exogenously added (L-Glu), the Raman I_1122_/I_1096_ intensity ratio of starch gels exhibited a non-monotonic change (Table S4), which reflects a concentration-dependent transition in the short-range molecular order of starch chains. At low concentrations, L-Glu may primarily act as an “interfering agent” that hinders the ordered rearrangement of starch molecular chains, resulting in reduced short-range molecular order. When the L-Glu concentration rises to a higher level, the rebound of the Raman ratio suggests the possible formation of new ordered structures in the system. Combined with multi-dimensional characterization results, this observation collectively supports the inference that L-Glu forms specific hydrogen-bonded complexes with leached amylose: after co-gelatinization of the L-Glu-starch mixture, a broadened diffraction peak appears in the XRD pattern of the starch gel, indicating the disruption of the native crystalline structure of starch and providing indirect evidence for the possible formation of starch-L-Glu complexes, which needs to be further verified by more targeted structural characterization (Xu et al., 2025); the significant elevation in gelatinization temperature from DSC measurements (Table 2) corresponds to the higher energy input required to disrupt hydrogen-bonded complexes, which thermodynamically corroborates the formation of stable complexes; meanwhile, the reduction in setback viscosity determined by RVA (Table 3) is consistent with the well-established pattern that starch-ligand complexes inhibit intermolecular rearrangement of starch molecules and exert anti-retrogradation effects. Taken together, via the dual pathways of inhibiting the formation of conventional starch-starch double helices and simultaneously inducing the generation of starch-glutamate complexes, L-Glu collectively contributes to the weakened network structure and enhanced anti-retrogradation performance of starch gels.

## 5 Conclusions

This study elucidates the comprehensive mechanism by which L-Glu affects wheat starch, spanning from field metabolic regulation to in vitro structural modification. The results demonstrate that foliar application of exogenous L-Glu optimizes the metabolic background for starch biosynthesis under drought stress by reshaping grain carbon and nitrogen metabolic profiles. When added in vitro, L-Glu acts as an efficient starch physicochemical property improver, and molecular dynamics simulations confirmed its molecular mechanism: L-Glu forms 1.47–2.18 stable hydrogen bonds with starch chains per system (total interaction energy, −169.3 to −205.3 kJ/mol), thereby directly intervening in the starch matrix. This effect is concentration dependent. With increasing L-Glu concentration, the RMSD of the starch single helix increases, and the dihedral-angle distribution of the α-1,4-glycosidic bond broadens. In addition, in vitro characterization experiments showed that the short-range ordered structure of starch is disrupted, leading to enhanced in vitro digestibility and altered gelatinization behavior and starch gel network strength. Moreover, by competing for hydration sites and introducing steric hindrance and electrostatic repulsion, L-Glu effectively inhibits starch chain rearrangement and crystallization, exhibiting potential for suppressing retrogradation. Overall, this study provides mechanistic insights into amino acid-starch interactions that hold potential for mitigating starch retrogradation and developing fresh, ready-to-eat wheat-based products, offering a mild and tunable strategy for improving starch quality.

## CRediT authorship contribution statement

**Gang Li:** Writing – review & editing, Writing – original draft, Visualization, Validation, Supervision, Software, Resources, Methodology, Investigation, Formal analysis, Data curation, Conceptualization. **Song Shen:** Methodology, Formal analysis, Data curation, Conceptualization. **Ke Wang:** Methodology, Investigation, Formal analysis, Data curation, Conceptualization. **Kaiyong Fu:** Formal analysis, Data curation, Conceptualization. **Jialian Wei:** Investigation, Formal analysis, Data curation, Conceptualization. **Heqin Wei:** Investigation, Formal analysis, Data curation, Conceptualization. **Xun Zhu:** Formal analysis, Data curation, Conceptualization. **Chunyan Li:** Writing – review & editing, Writing – original draft, Visualization, Validation, Supervision, Software, Resources, Project administration, Methodology, Investigation, Funding acquisition, Formal analysis, Data curation, Conceptualization. **Cheng Li:** Visualization, Validation, Supervision, Software, Resources, Project administration, Methodology, Investigation, Funding acquisition, Formal analysis, Data curation, Conceptualization.

## Author statement

The authors declare that they have not used AI to produce this manuscript.

## Declaration of competing interest

The authors declare that they have no known competing financial interests or personal relationships that could have appeared to influence the work reported in this paper.

## Data Availability

Data will be made available on request.
