# Supplementary material for: Effects of L-glutamic acid on wheat starch structure and functionality: multiscale characterization and molecular dynamics simulations
Source: Food Chem X. 2026 Jul 31;38:104267. doi: 10.1016/j.fochx.2026.104267 (PMC13475660; doi:10.1016/j.fochx.2026.104267)
Supplement: Supplementary file 1 [file mmc1.docx]

**Supplementary Materials**

Fig. S1 Enrichment analysis and correlation analysis of differential metabolites in grains under drought stress after post-anthesis

A, B: Enrichment analysis of differential substances; C, D: Correlation analysis of differential substances.

Fig. S2 Effect of L-Glu on enzymatic hydrolysis of starch granules under post-anthesis drought stress

WT_G0: Well Watered Treatment + 0 mmol/L L-Glu; DT_G0: Drought Treatment + 0 mmol/L L-Glu; DT18_G0: XD18 Drought Treatment + 0 mmol/L L-Glu (field); DT22_G0: XD22 Drought Treatment + 0 mmol/L L-Glu (field); A-D: Laser confocal microscopy observation of starch granules (CLSM Results of Newly Extracted Amyl Particle Observations, consistent with our group's previous findings, Li et al., 2025); E, F: Reducing sugar concentration measurement results.

Fig. S3 Effects of L-Glu addition on the physicochemical properties of starch samples

DT18_G0: XD18 drought treatment + 0 mmol/L L-Glu (field); DT22_G0: XD22 drought treatment + 0 mmol/L L-Glu (field); 0%-DT18_G0: addition of 0% L-Glu to starch from DT18_G0; 3.75%-DT18_G0: addition of 3.75% L-Glu to starch from DT18_G0; 7.5%-DT18_G0: addition of 7.5% L-Glu to starch from DT18_G0; 0%-DT22_G0: addition of 0% L-Glu to starch from DT22_G0; 3.75%-DT22_G0: addition of 3.75% L-Glu to starch from DT22_G0; 7.5%-DT22_G0: addition of 7.5% L-Glu to starch from DT22_G0.

Fig. S4 Schematic diagram of glutamic acid structure (A) and aqueous starch solution system (B)

Fig. S5 Schematic molecular structure of the α-1,4-glycosidic bond

Table S1 pH value and conductivity of amino acid and starch blend emulsion

| starch | Glu Contents/% | pH | Electrical conductivity  μs/cm |
| --- | --- | --- | --- |
| DT18_G0 | 0 | 7.21 | 31.00 |
|  | 3.75 | 3.84 | 136.40 |
|  | 7.5 | 3.59 | 178.00 |
| DT22_G0 | 0 | 7.10 | 27.60 |
|  | 3.75 | 3.64 | 137.70 |
|  | 7.5 | 3.47 | 181.30 |

Notes: DT18_G0: XD18 Drought Treatment + 0 mmol/L L-Glu (field); DT22_G0: XD22 Drought Treatment + 0 mmol/L L-Glu (field).

Table S2 Influence of different concentrations of L-Glu on Fourier infrared spectrum 1047/1022 and 1022/995 of starch samples

| starch | Glu Contents/% | 1047/1022 | 1022/995 |
| --- | --- | --- | --- |
| DT18_G0 | 0 | 1.12 | 0.91 |
|  | 3.75 | 1.26 | 0.94 |
|  | 7.5 | 1.16 | 0.90 |
| DT22_G0 | 0 | 1.19 | 0.97 |
|  | 3.75 | 1.40 | 0.93 |
|  | 7.5 | 1.20 | 0.90 |

Notes: DT18_G0: XD18 Drought Treatment + 0 mmol/L L-Glu (field); DT22_G0: XD22 Drought Treatment + 0 mmol/L L-Glu (field).

Table S3 Effect of different concentrations of L-Glu on Raman spectrum FWHM 480cm^-1^ of starch samples

| starch | Glu Contents/% | 480cm^-1^（Yp） | 480cm^-1^ FWHM |
| --- | --- | --- | --- |
| DT18_G0 | 0 | 110.90 | 14.72 |
|  | 3.75 | 105.42 | 15.31 |
|  | 7.5 | 66.46 | 15.80 |
| DT22_G0 | 0 | 92.81 | 13.52 |
|  | 3.75 | 83.83 | 12.70 |
|  | 7.5 | 73.99 | 15.05 |

Notes: DT18_G0: XD18 Drought Treatment + 0 mmol/L L-Glu (field); DT22_G0: XD22 Drought Treatment + 0 mmol/L L-Glu (field).

Table S4 Effects of Different Concentrations of L-Glu on Variations in the Raman Spectral Intensity Ratio I_1122_/I_1096_ of Starch Samples (Starch Gel)

| starch | Glu Contents/% | I_1122_/I_1096_ |
| --- | --- | --- |
| DT18_G0 | 0 | 0.998 |
|  | 3.75 | 0.963 |
|  | 7.5 | 1.011 |
| DT22_G0 | 0 | 1.001 |
|  | 3.75 | 0.995 |
|  | 7.5 | 1.007 |

Notes: DT18_G0: XD18 Drought Treatment + 0 mmol/L L-Glu (field); DT22_G0: XD22 Drought Treatment + 0 mmol/L L-Glu (field).

Table S5 Partial Differential Metabolic Name Table

| ID | Name |
| --- | --- |
| MP19209 | 11,12-DiHETrE-EA |
| MP14471 | -pinene oxide |
| MP15500 | (2z,6e,8r,10e,14z)-2,6,10,14-tetramethylhexadeca-2,6,10,14-tetraene-1,8,16-triol |
| MP20236 | 2-[2-(azepan-1-yl)-2-oxoethyl]-1-hydroxy-1-phenyl-decahydropyrido[1,2-a]azepin-4-one |
| MP7390 | vomifoliol |
| MP15384 | 3-Methyl-5-[(1S,2R,4aR)-1,2,4a,5-tetramethyl-7-oxo-1,2,3,4,4a,7,8,8a-octahydro-1-naphthalenyl]pentanoic acid |
| MP19146 | 5beta-pregnane-11beta,17alpha,20alpha,21-tetrol-3-one |
| MP15381 | (3S)-5-[(4aR,8aS)-2,5,5,8a-Tetramethyl-3-oxo-4a,6,7,8-tetrahydro-4H-naphthalen-1-yl]-3-methylpentanoic acid |
| MP19257 | (3aR,3a1S,5S,5aS,10bS)-methyl 6-acetyl-3a-ethyl-2,3,3a,3a1,4,5,5a,6,11,12-decahydro-1H-indolizino[8,1-cd]carbazole-5-carboxylate |
| MP23715 | (1s,1's,2r,6s,6's,7's,9's,11'r)-6,11'-bis(acetyloxy)-7'-hydroxy-3,3-dimethyl-10'-methylidene-2'-oxo-3'-oxaspiro[cyclohexane-1,5'-tricyclo[7.2.1.0,]dodecane]-2-carboxylic acid |
| MP19551 | Pyraclostrobin |
| MP24724 | Forsythoside E |
| MP19500 | (3E)-4-[2-(Hydroxymethyl)-6,6-dimethyl-4-oxo-2-cyclohexen-1-yl]-3-buten-2-yl beta-D-glucopyranoside |
| MP36941 | [(2R,3S,4S,5R,6R)-3,4,5-trihydroxy-6-{[(2S,3S,4R,5R)-4-hydroxy-3-{[(2E)-3-(4-hydroxy-3,5-dimethoxyphenyl)prop-2-enoyl]oxy}-2,5-bis(hydroxymethyl)oxolan-2-yl]oxy}oxan-2-yl]methyl (2E)-3-(4-hydroxy-3,5-dimethoxyphenyl)prop-2-enoate |
| MP13724 | (8r)-8-hydroxy-2-nonyl-5,6,7,8-tetrahydrochromen-4-one |
| MP24191 | MGTS(16:0/0:0) |
| MP3941 | DNP-DL-Methionine |
| MP5023 | L-METHIONINE |
| MP2347 | L-Serine |
| MP18675 | CHOLATE |
